# Supplementary material for: Surface protein imputation from single cell transcriptomes by deep neural networks
Source: Nat Commun. 2020 Jan 31;11:651. doi: 10.1038/s41467-020-14391-0 (PMC6994606; doi:10.1038/s41467-020-14391-0)
Supplement: Supplementary file 1 — Supplementary Information [file 41467_2020_14391_MOESM1_ESM.pdf]

Surface protein imputation from single cell transcriptomes by deep neural networks

Zhou, et al.

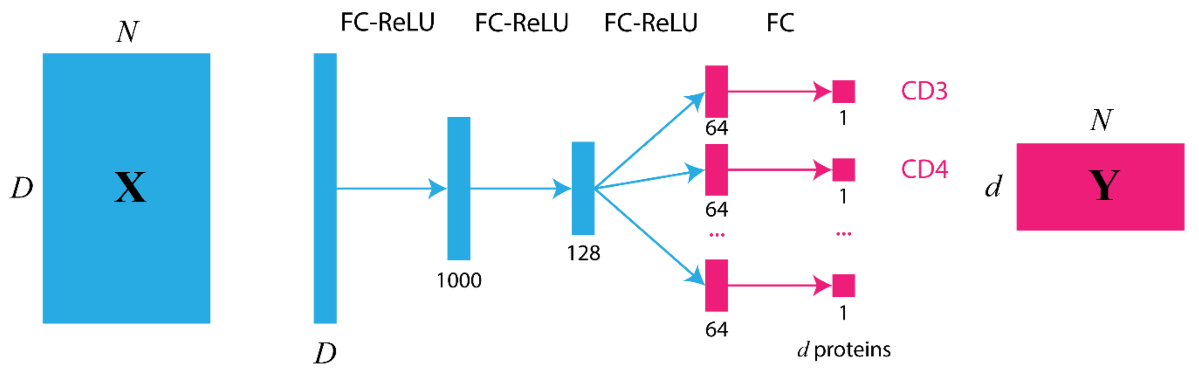

Supplementary Figure 1

Neural network architecture of the cTP-net.

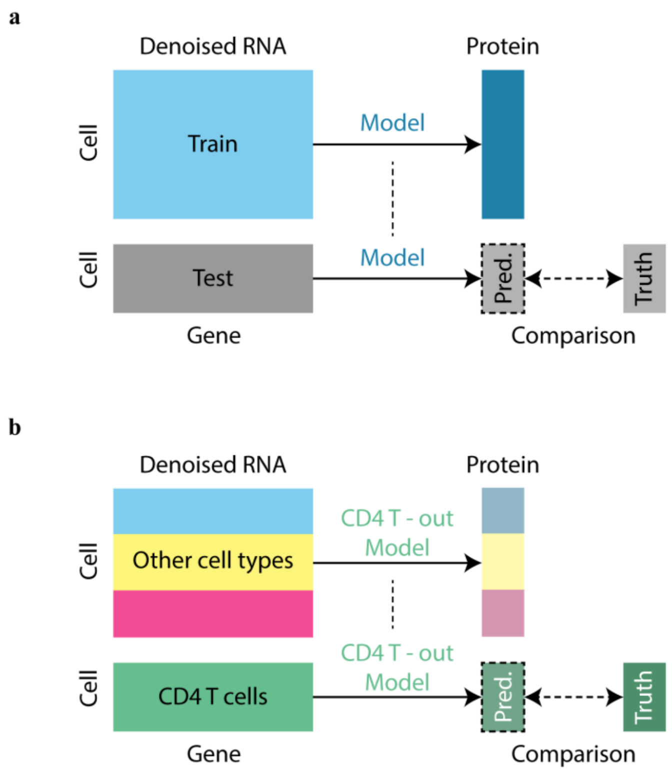

Supplementary Figure 2

Benchmark procedure.

(a) Holdout method validation scheme. (b) Out-of-cell-type benchmark scheme.

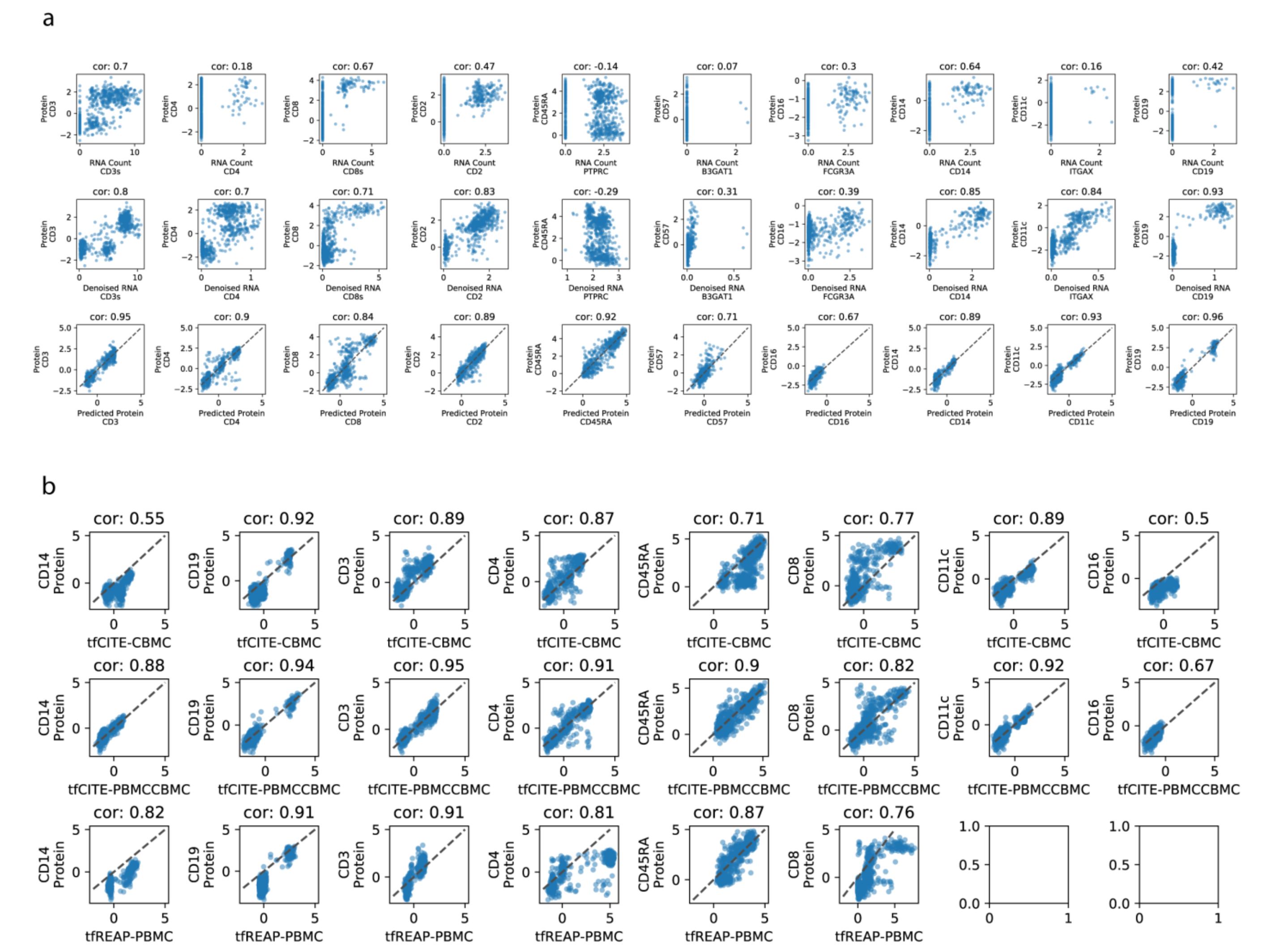
a
cor: 0.7
cor: 0.18
cor: 0.67
cor: 0.47
cor: -0.14
cor: 0.07
cor: 0.3
cor: 0.64
cor: 0.16
cor: 0.42
RNA Count CD3s
RNA Count CD4
RNA Count CD8s
RNA Count CD2
RNA Count PTPRC
RNA Count B3GAT1
RNA Count FCGR3A
RNA Count CD14
RNA Count ITGAX
RNA Count CD19
cor: 0.8
cor: 0.7
cor: 0.71
cor: 0.83
cor: -0.29
cor: 0.31
cor: 0.39
cor: 0.85
cor: 0.84
cor: 0.93
Denoised RNA CD3s
Denoised RNA CD4
Denoised RNA CD8s
Denoised RNA CD2
Denoised RNA PTPRC
Denoised RNA B3GAT1
Denoised RNA FCGR3A
Denoised RNA CD14
Denoised RNA ITGAX
Denoised RNA CD19
cor: 0.95
cor: 0.9
cor: 0.84
cor: 0.89
cor: 0.92
cor: 0.71
cor: 0.67
cor: 0.89
cor: 0.93
cor: 0.96
Predicted Protein CD3
Predicted Protein CD4
Predicted Protein CD8
Predicted Protein CD2
Predicted Protein CD45RA
Predicted Protein CD57
Predicted Protein CD16
Predicted Protein CD14
Predicted Protein CD11c
Predicted Protein CD19
b
cor: 0.55
cor: 0.92
cor: 0.89
cor: 0.87
cor: 0.71
cor: 0.77
cor: 0.89
cor: 0.5
CD14 Protein
CD19 Protein
CD3 Protein
CD4 Protein
CD45RA Protein
CD8 Protein
CD11c Protein
CD16 Protein
tfCITE-CBMC
cor: 0.88
cor: 0.94
cor: 0.95
cor: 0.91
cor: 0.9
cor: 0.82
cor: 0.92
cor: 0.67
tfCITE-PBMCCBMC
cor: 0.82
cor: 0.91
cor: 0.91
cor: 0.81
cor: 0.87
cor: 0.76
tfREAP-PBMC

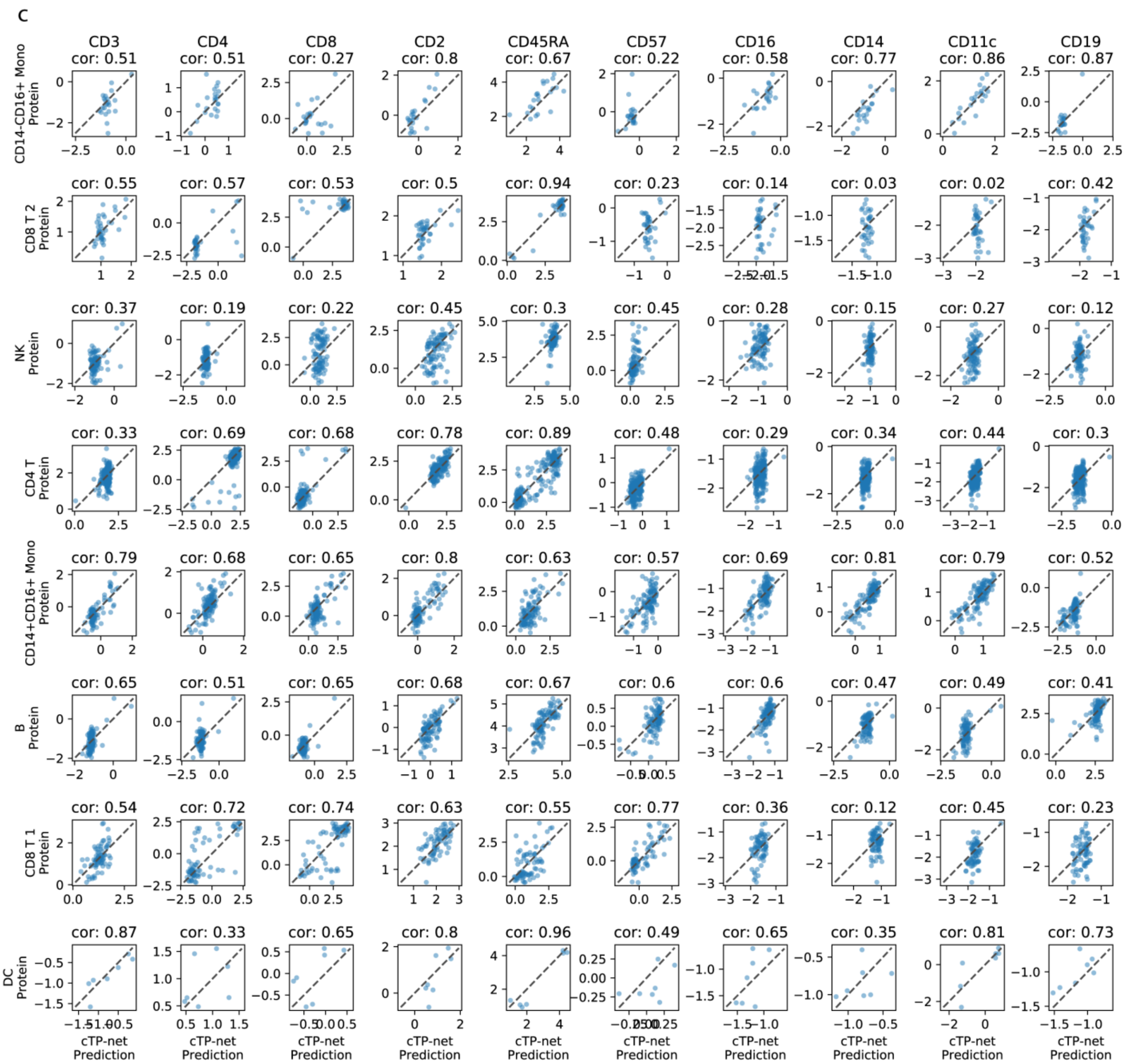

Supplementary Figure 3

Benchmark evaluation of cTP-net on CITE-PBMC data set.

(a) Benchmark correlation of true protein level vs. (1) Raw RNA count, (2) SAVER-X denoised RNA level, and (3) cTP-net predicted protein abundance in holdout method. (b) Benchmark correlation of truth protein level vs. (1) transfer learning from CITE-CBMC, (2) transfer learning from CITE-PBMCCBMC, and (3) transfer learning from REAP-PBMC. (c) Benchmark correlation of true protein level vs. cTP-net prediction in holdout method for each cell type.

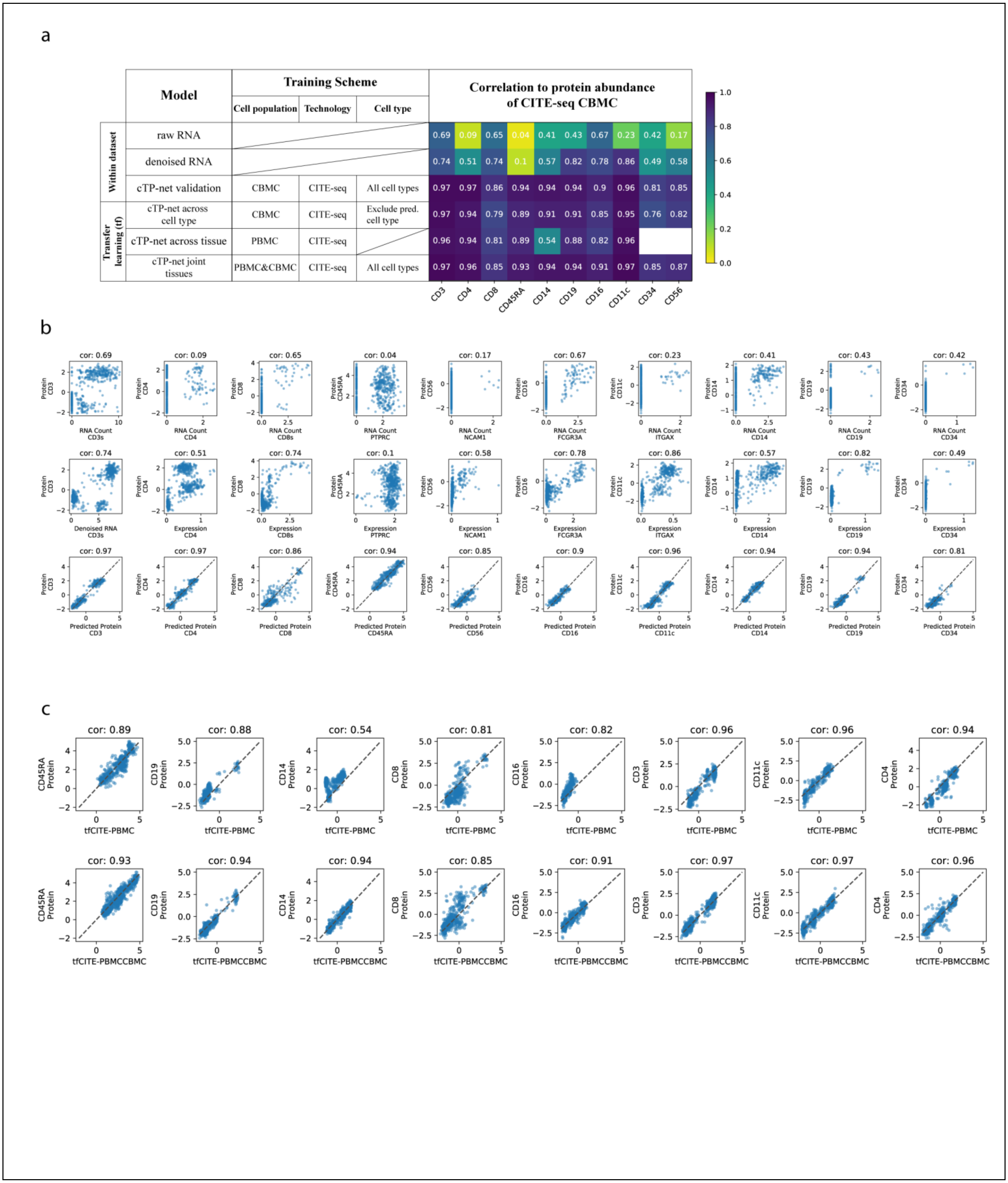
a
Model
Training Scheme
Cell population
Technology
Cell type
Correlation to protein abundance of CITE-seq CBMC
Within dataset
Transfer learning (tf)
raw RNA
denoised RNA
cTP-net validation
cTP-net across cell type
cTP-net across tissue
cTP-net joint tissues
CBMC
PBMC
PBMC&CBMC
CITE-seq
All cell types
Exclude pred. cell type
0.69 0.09 0.65 0.04 0.41 0.43 0.67 0.23 0.42 0.17
0.74 0.51 0.74 0.1 0.57 0.82 0.78 0.86 0.49 0.58
0.97 0.97 0.86 0.94 0.94 0.94 0.9 0.96 0.81 0.85
0.97 0.94 0.79 0.89 0.91 0.91 0.85 0.95 0.76 0.82
0.96 0.94 0.81 0.89 0.54 0.88 0.82 0.96
0.97 0.96 0.85 0.93 0.94 0.94 0.91 0.97 0.85 0.87
CD3 CD4 CD8 CD45RA CD14 CD19 CD16 CD11c CD34 CD56
1.0 0.8 0.6 0.4 0.2 0.0
b
cor: 0.69 cor: 0.09 cor: 0.65 cor: 0.04 cor: 0.17 cor: 0.67 cor: 0.23 cor: 0.41 cor: 0.43 cor: 0.42
RNA Count CD3s, CD4, CD8s, PTPRC, NCAM1, FCGR3A, ITGAX, CD14, CD19, CD34
cor: 0.74 cor: 0.51 cor: 0.74 cor: 0.1 cor: 0.58 cor: 0.78 cor: 0.86 cor: 0.57 cor: 0.82 cor: 0.49
Denoised RNA CD3s
Expression CD4, CD8s, PTPRC, NCAM1, FCGR3A, ITGAX, CD14, CD19, CD34
cor: 0.97 cor: 0.97 cor: 0.86 cor: 0.94 cor: 0.85 cor: 0.9 cor: 0.96 cor: 0.94 cor: 0.94 cor: 0.81
Predicted Protein CD3, CD4, CD8, CD45RA, CD56, CD16, CD11c, CD14, CD19, CD34
Protein CD3, CD4, CD8, CD45RA, CD56, CD16, CD11c, CD14, CD19, CD34
c
cor: 0.89 cor: 0.88 cor: 0.54 cor: 0.81 cor: 0.82 cor: 0.96 cor: 0.96 cor: 0.94
tfCITE-PBMC
cor: 0.93 cor: 0.94 cor: 0.94 cor: 0.85 cor: 0.91 cor: 0.97 cor: 0.97 cor: 0.96
tfCITE-PBMCCBMC
CD45RA Protein, CD19 Protein, CD14 Protein, CD8 Protein, CD16 Protein, CD3 Protein, CD11c Protein, CD4 Protein

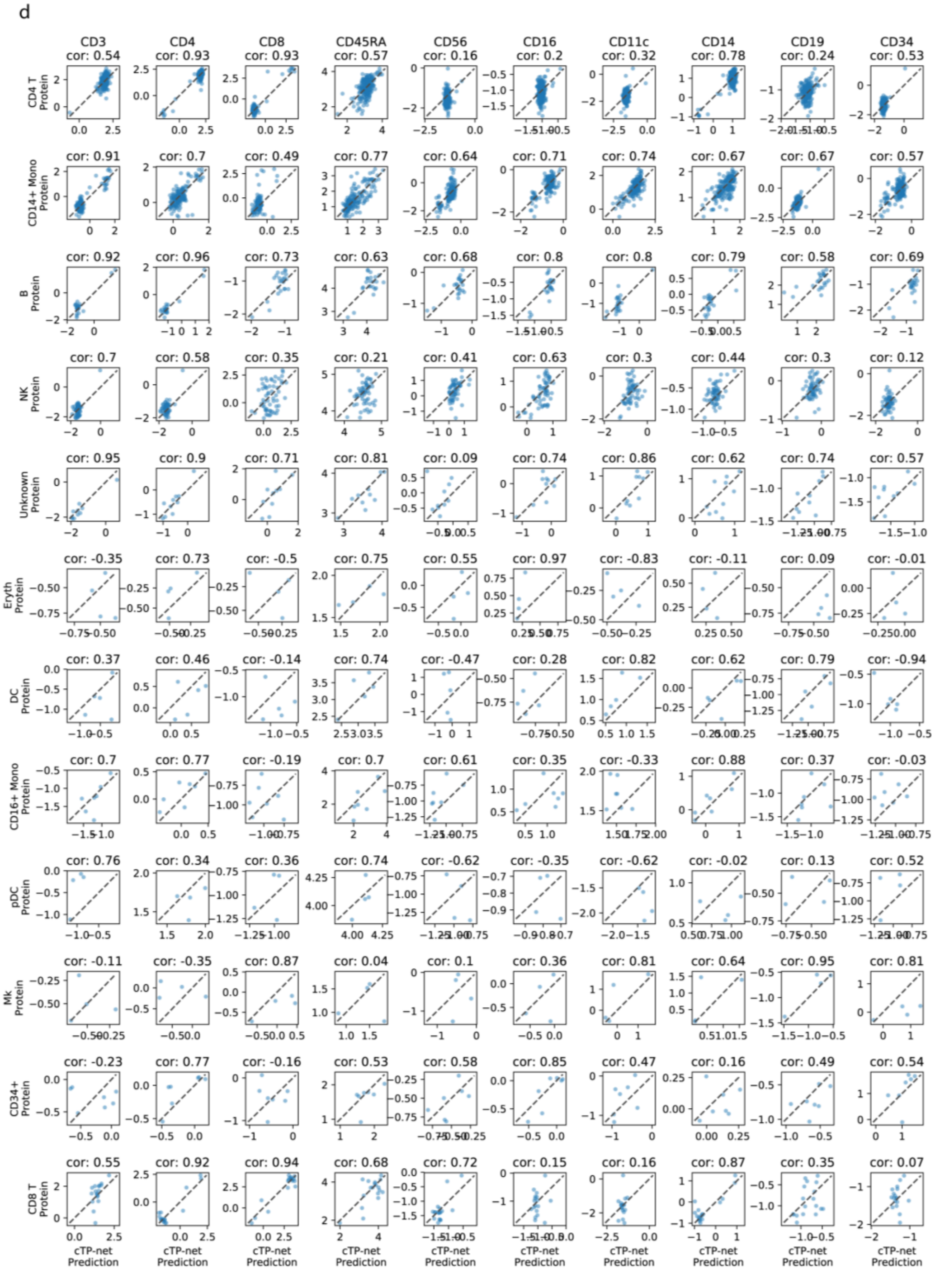

Supplementary Figure 4

Benchmark evaluation of cTP-net on CITE-CBMC data set.

(a) Benchmark evaluation heatmap of cTP-net and comparison with Seurat v3. The table on the left captures the detailed training scheme and model name of each test. **(b)** Benchmark correlation of true protein level vs. (1) Raw RNA count, (2) SAVER-X denoised RNA level, and (3) cTP-net predicted protein abundance in holdout method. **(c)** Benchmark correlation of truth protein level vs. (1) transfer learning from CITE-PBMC, and (2) transfer learning from CITE-PBMCCBMC. **(d)** Benchmark correlation of true protein level vs. cTP-net prediction in holdout method for each cell type.

a

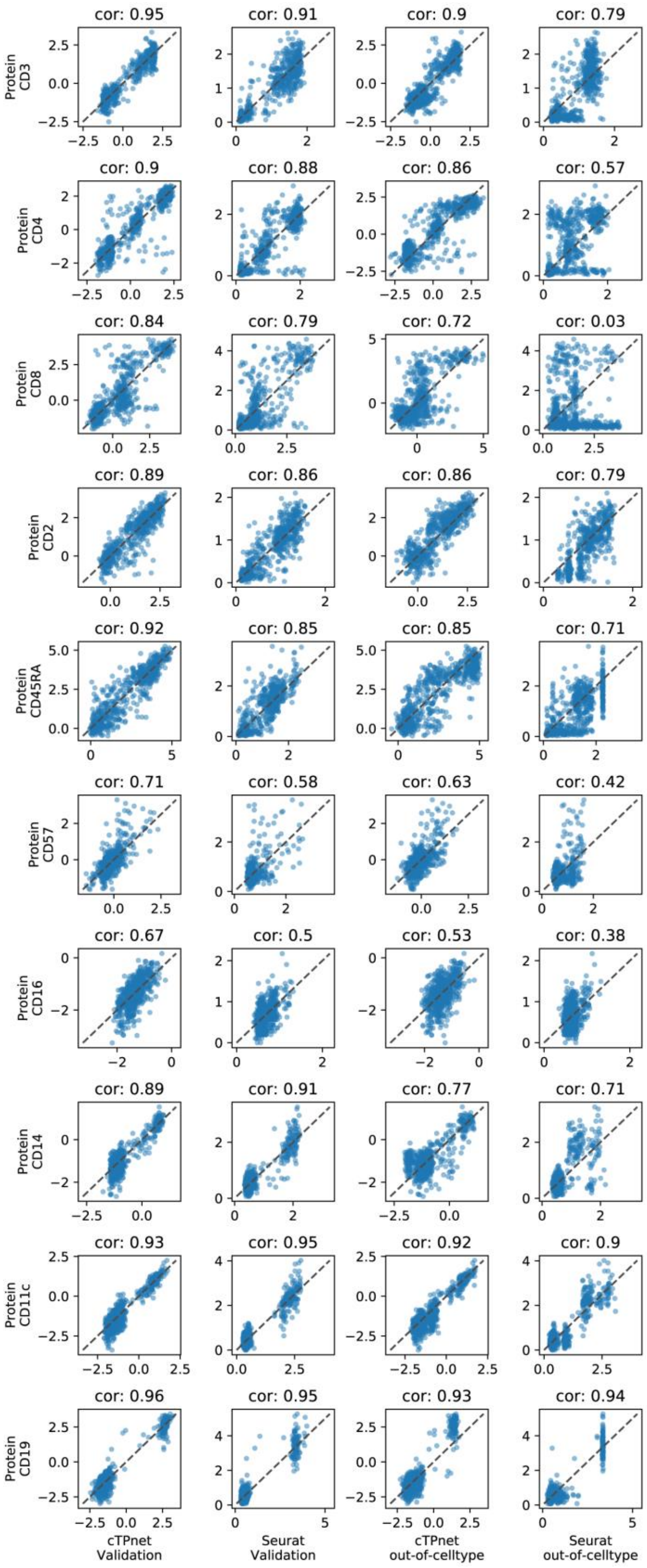
Protein CD3
cor: 0.95
cor: 0.91
cor: 0.9
cor: 0.79
Protein CD4
cor: 0.9
cor: 0.88
cor: 0.86
cor: 0.57
Protein CD8
cor: 0.84
cor: 0.79
cor: 0.72
cor: 0.03
Protein CD2
cor: 0.89
cor: 0.86
cor: 0.86
cor: 0.79
Protein CD45RA
cor: 0.92
cor: 0.85
cor: 0.85
cor: 0.71
Protein CD57
cor: 0.71
cor: 0.58
cor: 0.63
cor: 0.42
Protein CD16
cor: 0.67
cor: 0.5
cor: 0.53
cor: 0.38
Protein CD14
cor: 0.89
cor: 0.91
cor: 0.77
cor: 0.71
Protein CD11c
cor: 0.93
cor: 0.95
cor: 0.92
cor: 0.9
Protein CD19
cor: 0.96
cor: 0.95
cor: 0.93
cor: 0.94
cTPnet Validation
Seurat Validation
cTPnet out-of-celltype
Seurat out-of-celltype

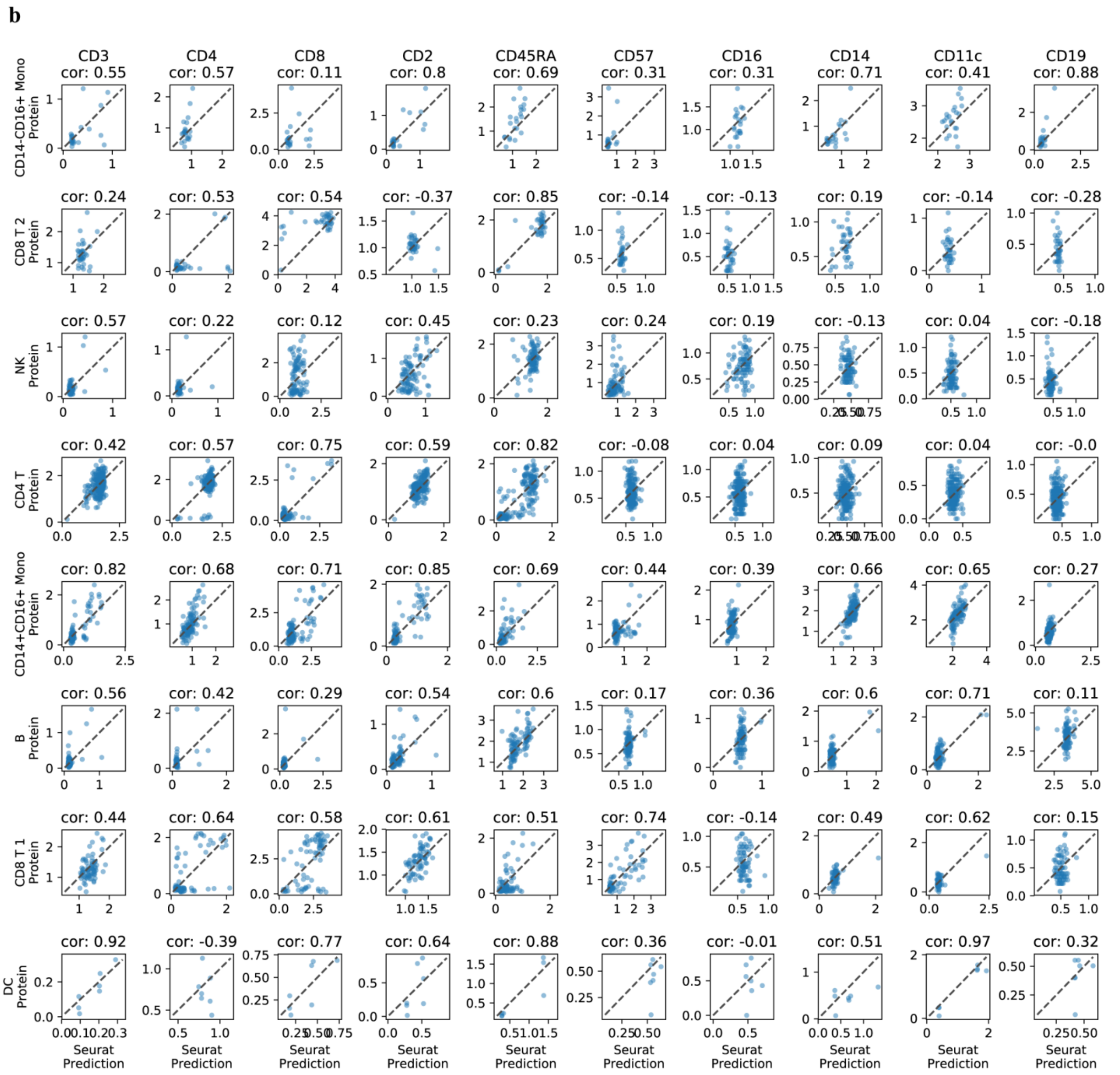

Supplementary Figure 5

Benchmark evaluation of Seurat v3 on CITE-PBMC data set.

(a) Benchmark correlation of true protein level vs. (1) cTP-net predicted protein abundance in holdout method, (2) Seurat v3 predicted protein abundance in holdout method, (3) out-of-cell-type cTP-net predicted protein abundance, and (4) out-of-cell-type Seurat v3 predicted protein abundance. **(b)** Benchmark correlation of truth protein level vs. (1) transfer learning from CITE-PBMC, and (2) transfer learning from CITE-PBMCCBMC. **(c)** Benchmark correlation of true protein level vs. cTP-net prediction in holdout method for each cell type.

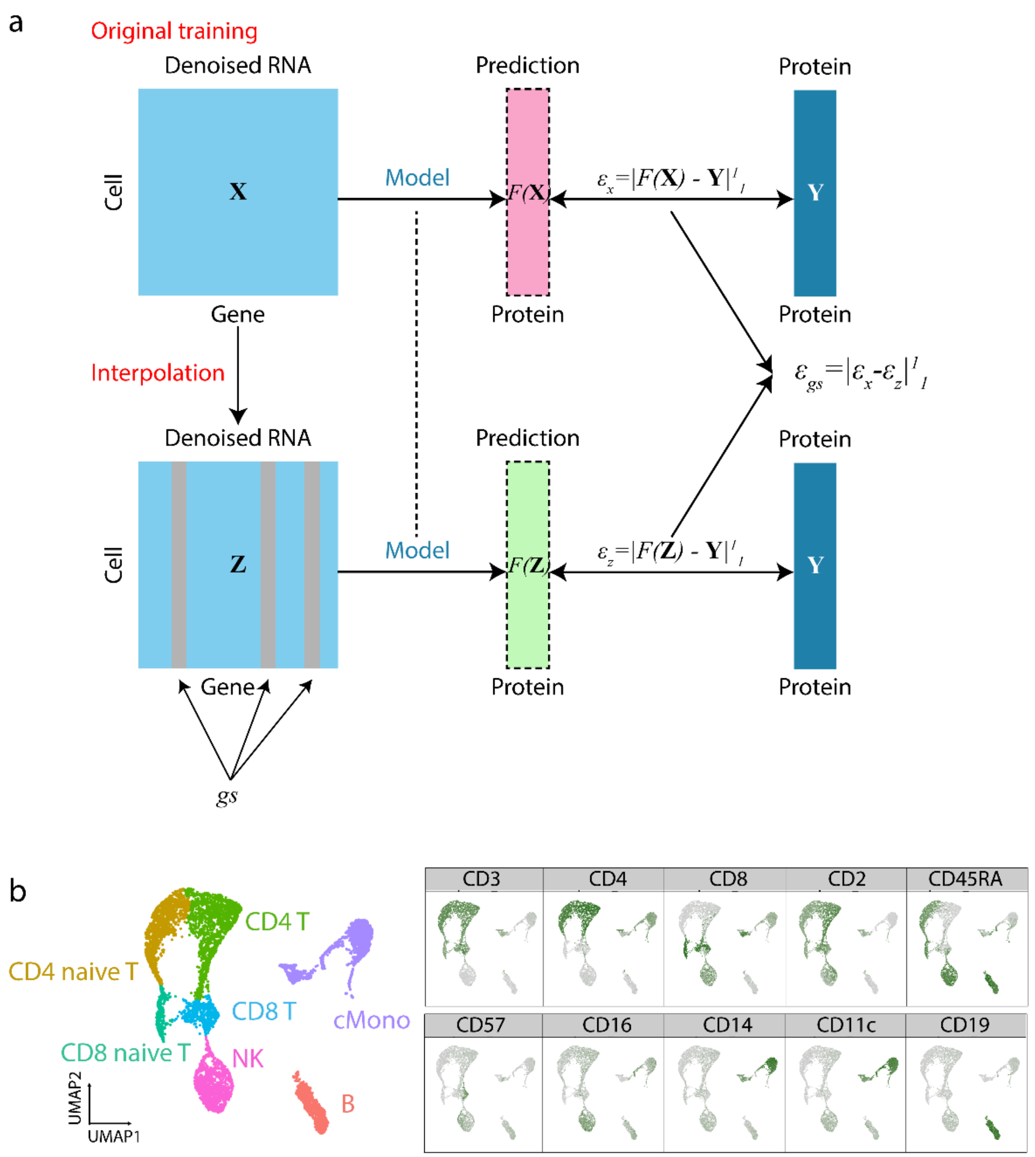

Supplementary Figure 6

Interpolation analysis.

(a) Interpolation procedure in identify permutation based importance score for each gene in each protein prediction. **(b)** Dimension reduction analysis on the bottleneck layer on cTP-net trained on PBMCs from CITE-seq.

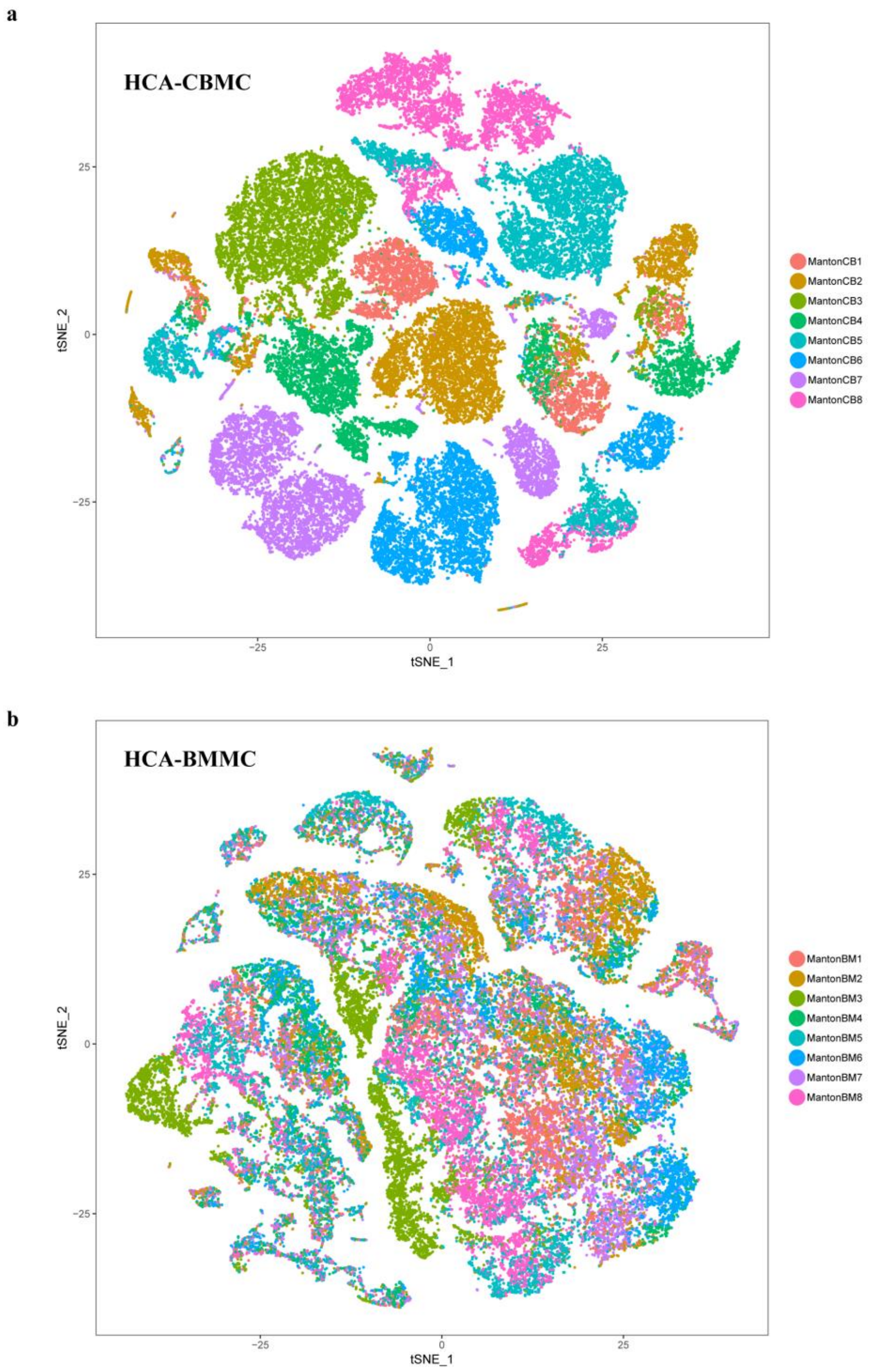

Supplementary Figure 7

Human Cell Atlas t-SNE plot based on normalized expression.

(a) t-SNE plot on Human Cell Atlas CBMCs based on normalized expression. Color indicates sample IDs. **(b)** t-SNE plot on Human Cell Atlas BMMCs based on normalized expression. Color indicates sample IDs. Strong batch effects observed in both data sets.

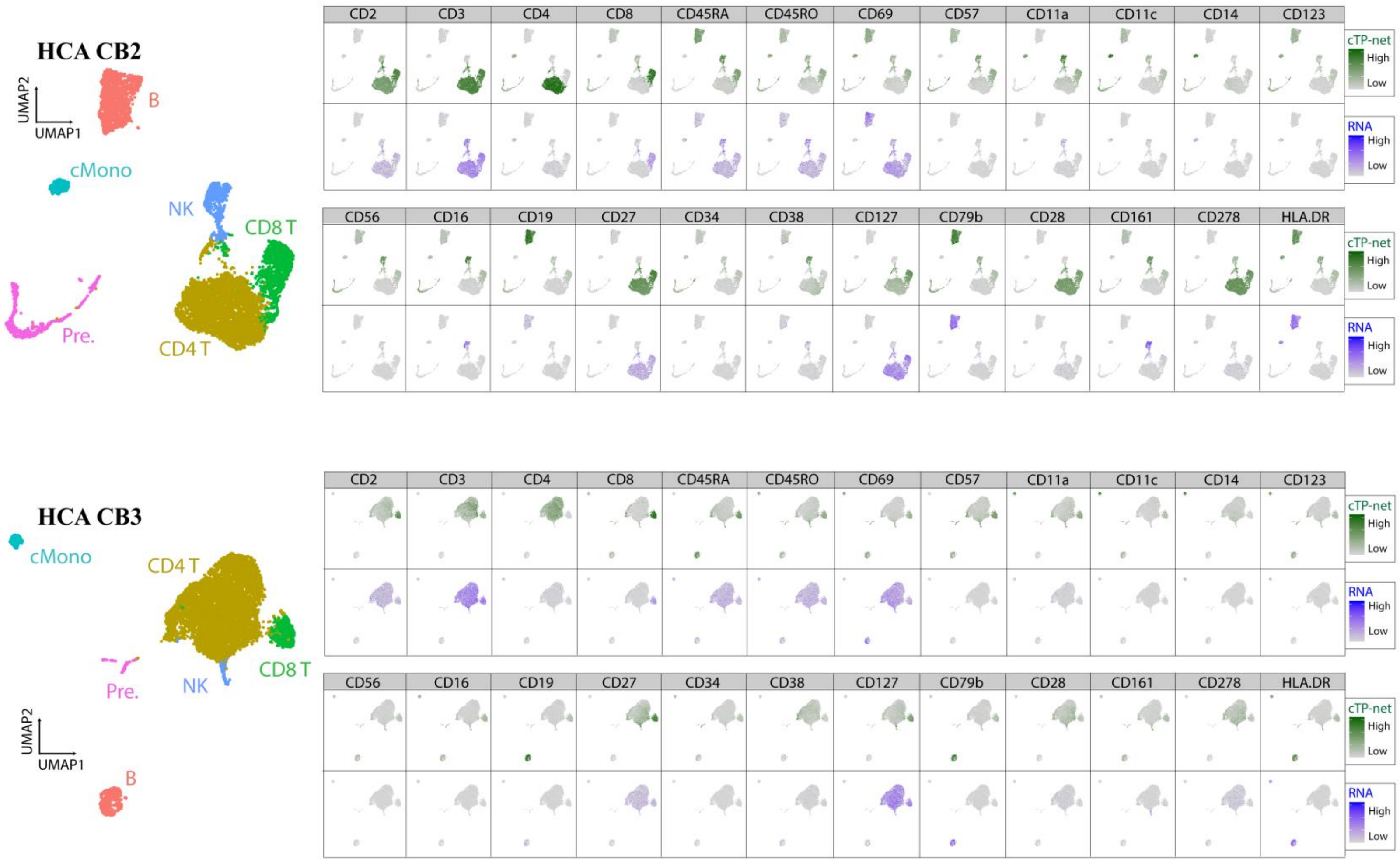

HCA CB2
UMAP2
UMAP1
B
cMono
NK
CD8 T
CD4 T
Pre.
CD2
CD3
CD4
CD8
CD45RA
CD45RO
CD69
CD57
CD11a
CD11c
CD14
CD123
CD56
CD16
CD19
CD27
CD34
CD38
CD127
CD79b
CD28
CD161
CD278
HLA.DR
cTP-net
High
Low
RNA
High
Low
HCA CB3
cMono
CD4 T
CD8 T
NK
Pre.
B
UMAP2
UMAP1

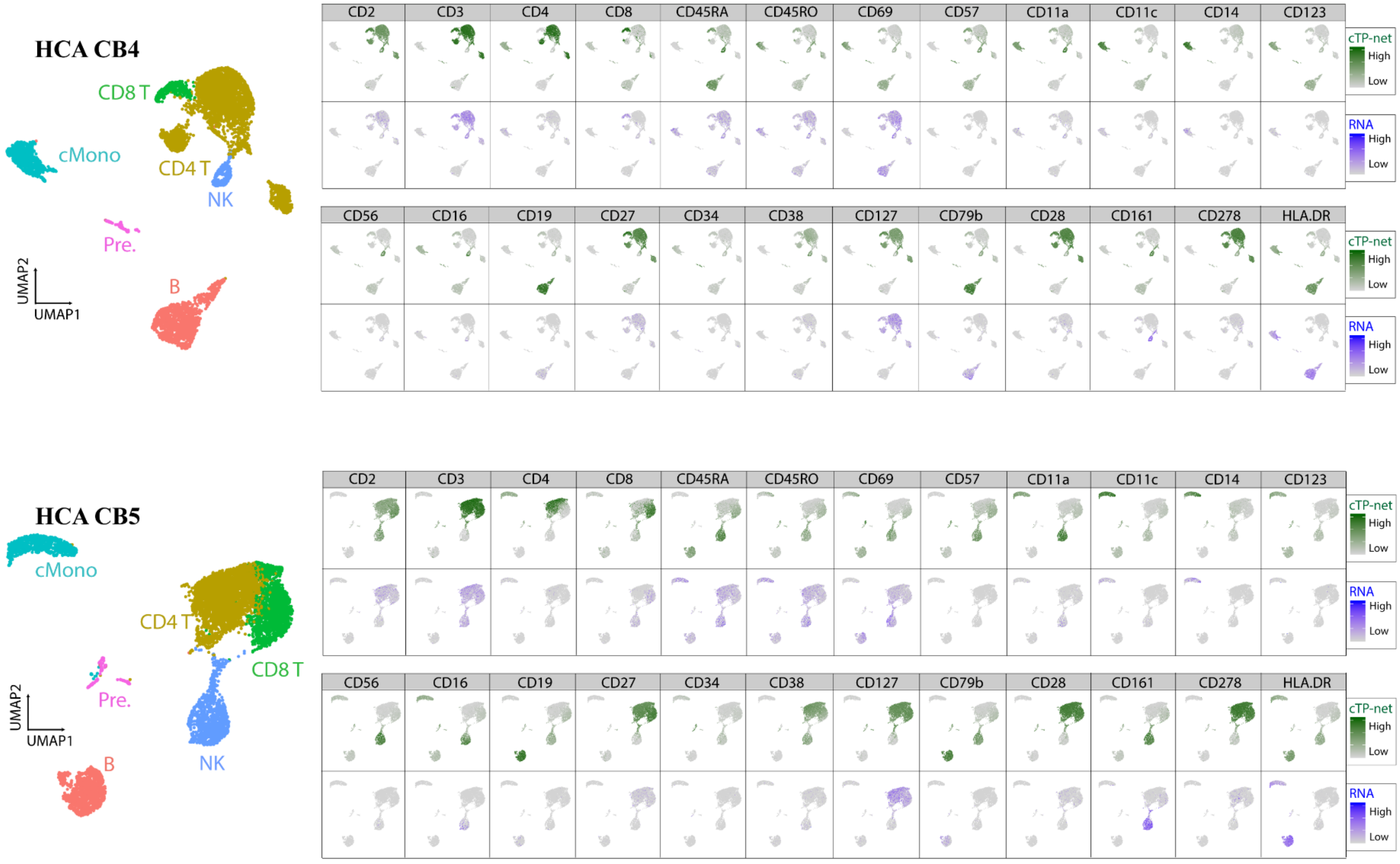
HCA CB4
CD8 T
cMono
CD4 T
NK
Pre.
B
UMAP2
UMAP1
CD2
CD3
CD4
CD8
CD45RA
CD45RO
CD69
CD57
CD11a
CD11c
CD14
CD123
CD56
CD16
CD19
CD27
CD34
CD38
CD127
CD79b
CD28
CD161
CD278
HLA.DR
cTP-net
High
Low
RNA
High
Low
HCA CB5
cMono
CD4 T
CD8 T
Pre.
NK
B
UMAP2
UMAP1

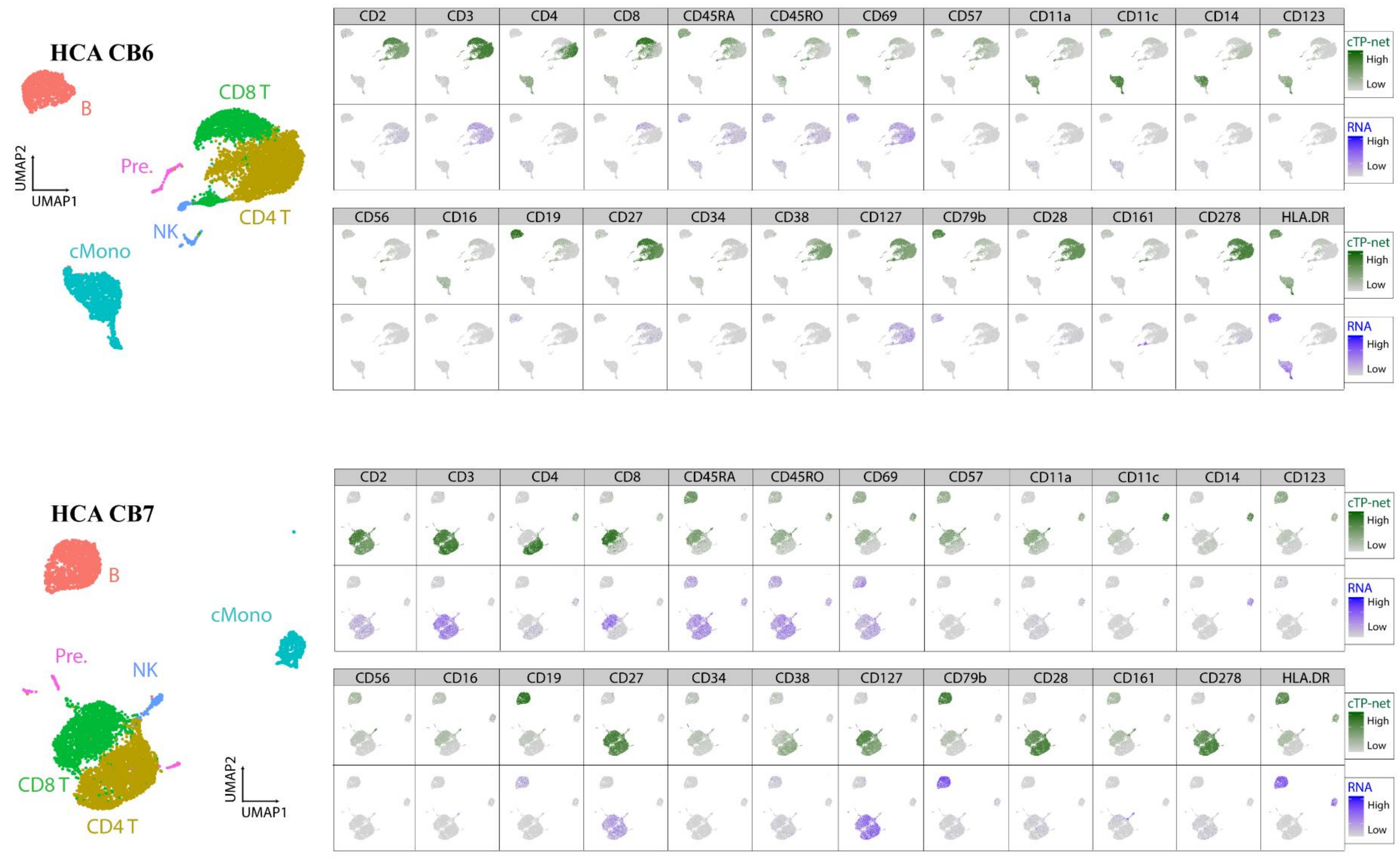

HCA CB6
B
CD8 T
Pre.
CD4 T
NK
cMono
UMAP2
UMAP1
CD2
CD3
CD4
CD8
CD45RA
CD45RO
CD69
CD57
CD11a
CD11c
CD14
CD123
CD56
CD16
CD19
CD27
CD34
CD38
CD127
CD79b
CD28
CD161
CD278
HLA.DR
cTP-net
High
Low
RNA
High
Low
HCA CB7
B
cMono
Pre.
NK
CD8 T
CD4 T
UMAP2
UMAP1

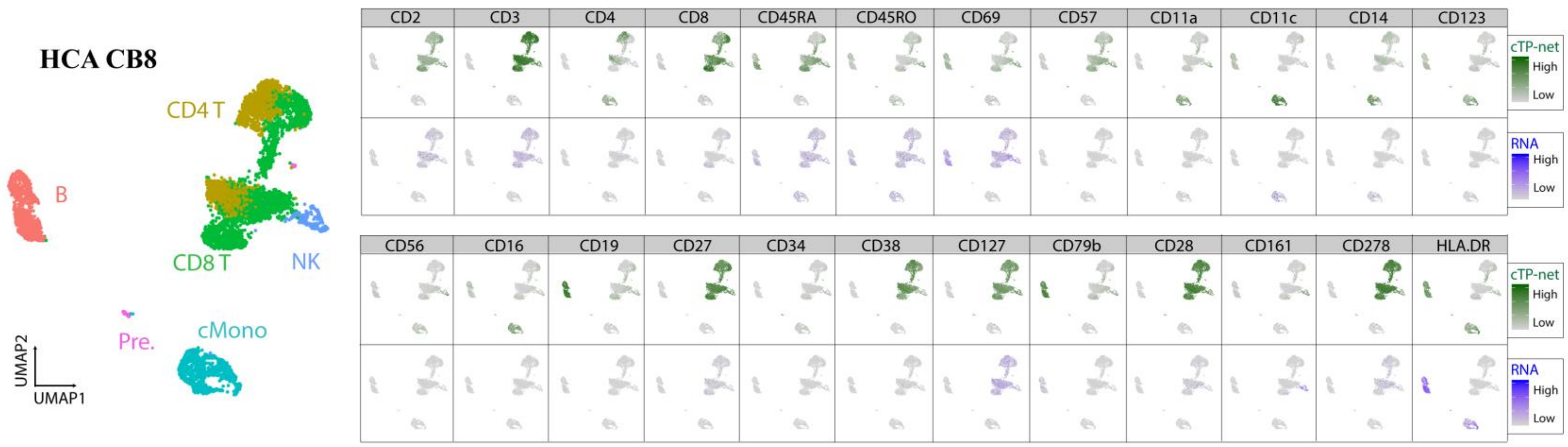
HCA CB8
CD4 T
B
CD8 T
NK
Pre.
cMono
UMAP2
UMAP1
CD2
CD3
CD4
CD8
CD45RA
CD45RO
CD69
CD57
CD11a
CD11c
CD14
CD123
cTP-net
High
Low
RNA
High
Low
CD56
CD16
CD19
CD27
CD34
CD38
CD127
CD79b
CD28
CD161
CD278
HLA.DR
cTP-net
High
Low
RNA
High
Low

Supplementary Figure 8
cTP-net prediction on Human Cell Atlas CBMCs by individual.
For each individual, we show (1) t-SNE visualization of HCA CBMCs based on expression. B: B cells; CD4 T: CD4 T cells; CD8 T: CD8 T cells; cMono: classic Monocyte; NK: Natural killer cells; Pre.: Precursors. (2) cTP-net imputed protein abundance and RNA of its cognate gene across 24 different surface proteins.

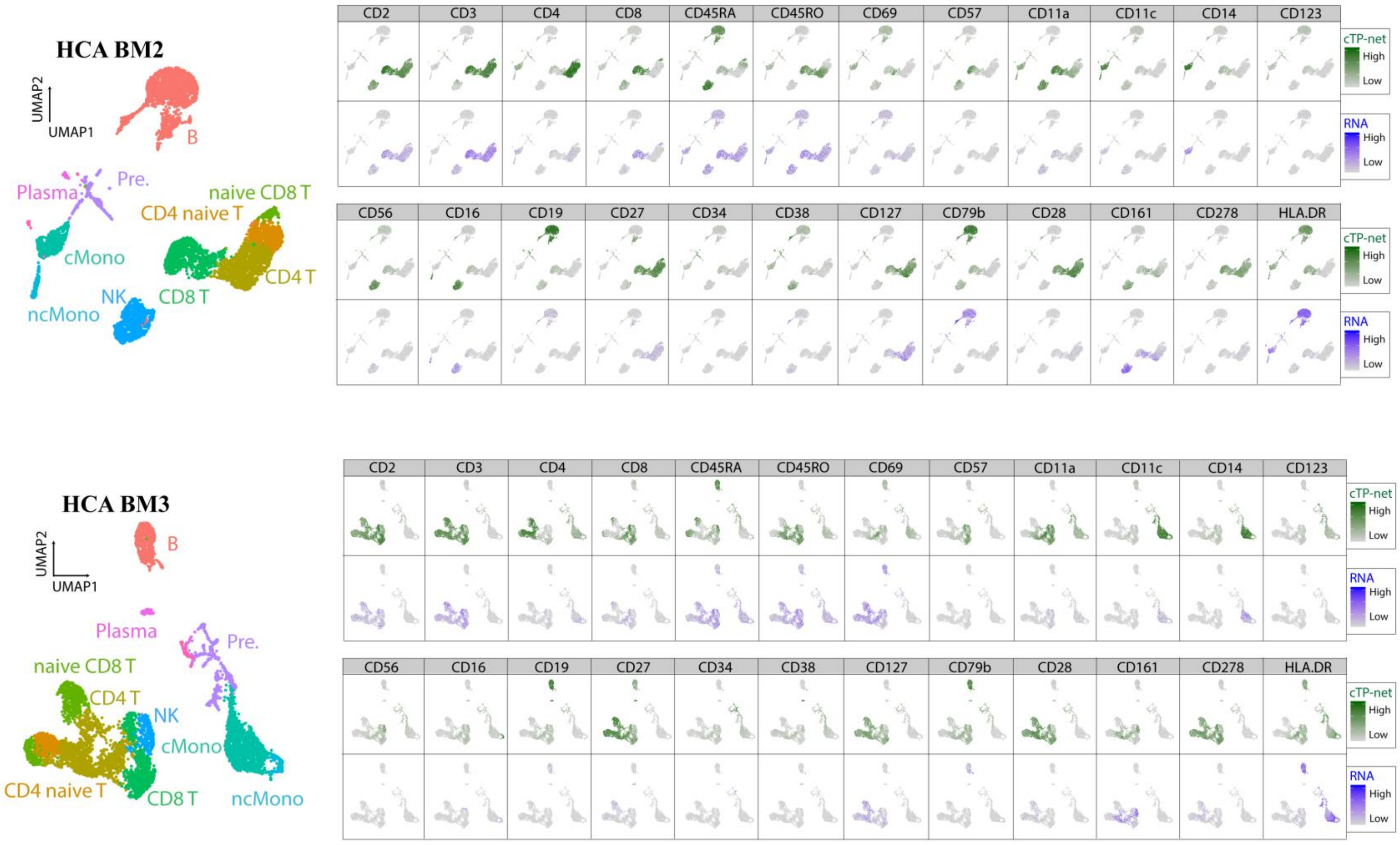
HCA BM2
UMAP2
UMAP1
B
Plasma
Pre.
naive CD8 T
CD4 naive T
cMono
CD4 T
NK
CD8 T
ncMono
CD2
CD3
CD4
CD8
CD45RA
CD45RO
CD69
CD57
CD11a
CD11c
CD14
CD123
CD56
CD16
CD19
CD27
CD34
CD38
CD127
CD79b
CD28
CD161
CD278
HLA.DR
cTP-net
High
Low
RNA
High
Low
HCA BM3
UMAP2
UMAP1
B
Plasma
Pre.
naive CD8 T
CD4 T
NK
cMono
CD4 naive T
CD8 T
ncMono

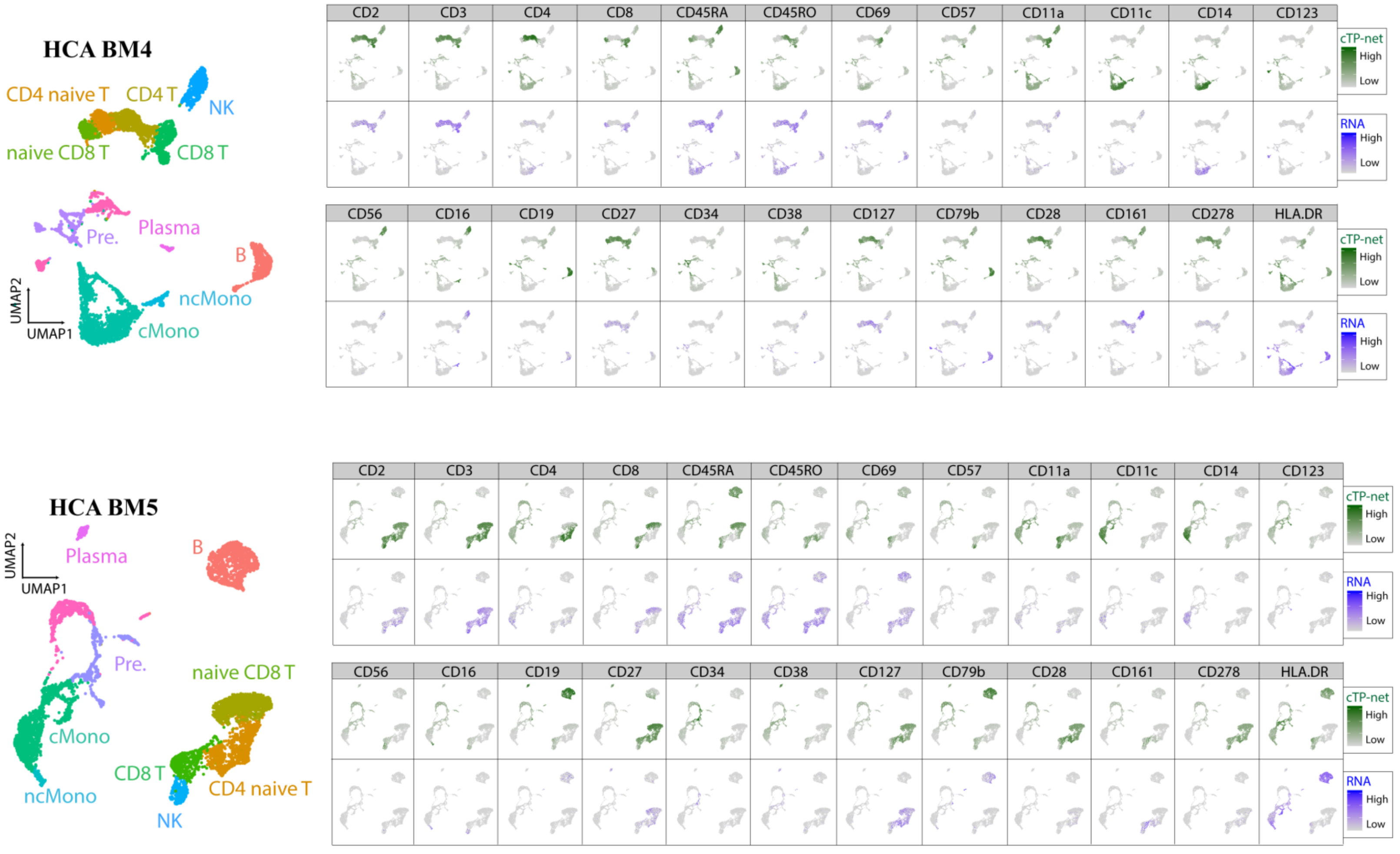
HCA BM4
CD4 naive T
CD4 T
NK
naive CD8 T
CD8 T
Pre.
Plasma
B
ncMono
cMono
UMAP2
UMAP1
CD2
CD3
CD4
CD8
CD45RA
CD45RO
CD69
CD57
CD11a
CD11c
CD14
CD123
CD56
CD16
CD19
CD27
CD34
CD38
CD127
CD79b
CD28
CD161
CD278
HLA.DR
cTP-net
RNA
High
Low
HCA BM5
Plasma
B
Pre.
naive CD8 T
cMono
CD8 T
CD4 naive T
ncMono
NK

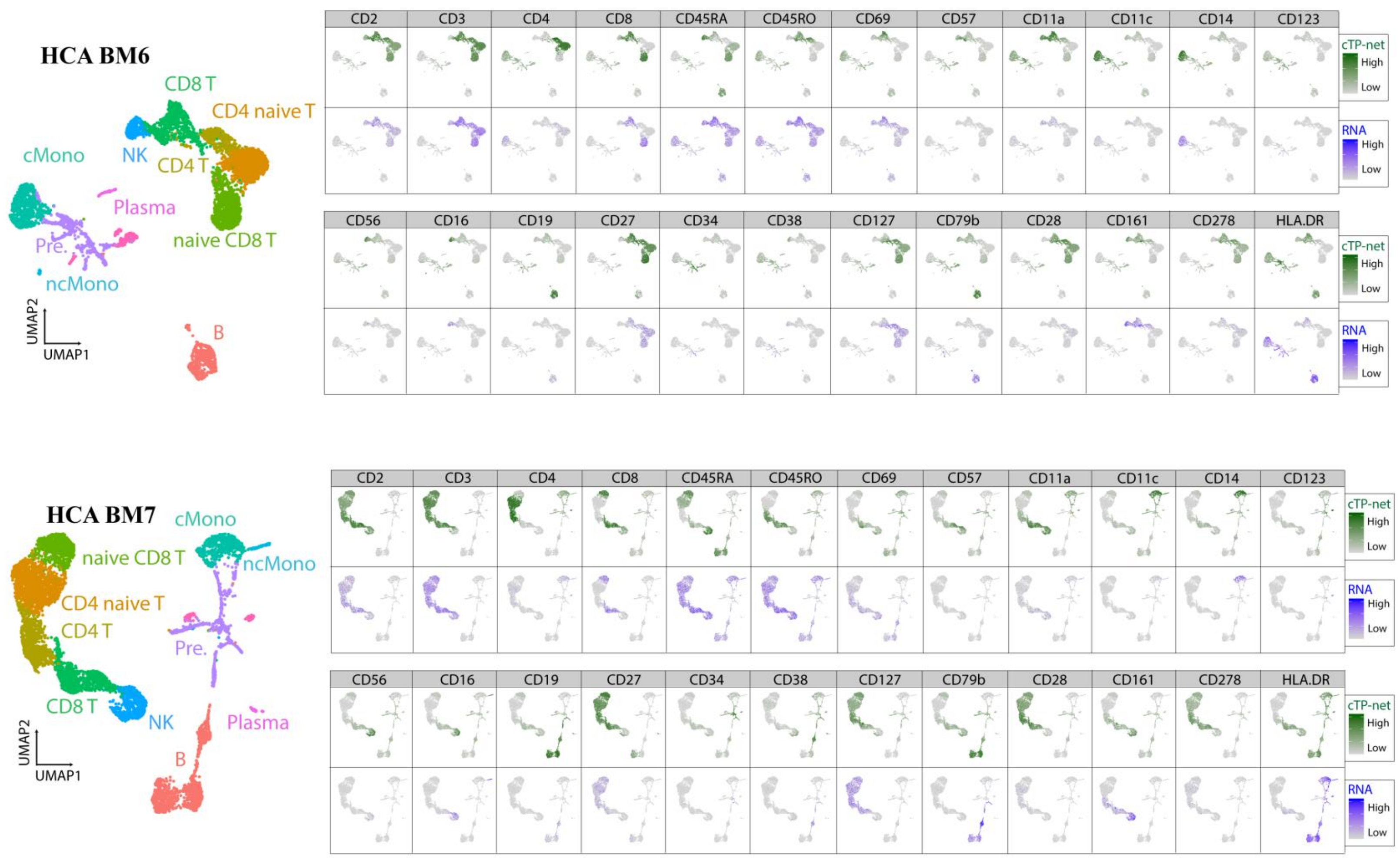
HCA BM6
CD8 T
CD4 naive T
NK
CD4 T
cMono
Plasma
Pre.
naive CD8 T
ncMono
B
UMAP2
UMAP1
CD2
CD3
CD4
CD8
CD45RA
CD45RO
CD69
CD57
CD11a
CD11c
CD14
CD123
CD56
CD16
CD19
CD27
CD34
CD38
CD127
CD79b
CD28
CD161
CD278
HLA.DR
cTP-net
High
Low
RNA
High
Low
HCA BM7
cMono
naive CD8 T
ncMono
CD4 naive T
CD4 T
Pre.
CD8 T
NK
Plasma
B
UMAP2
UMAP1

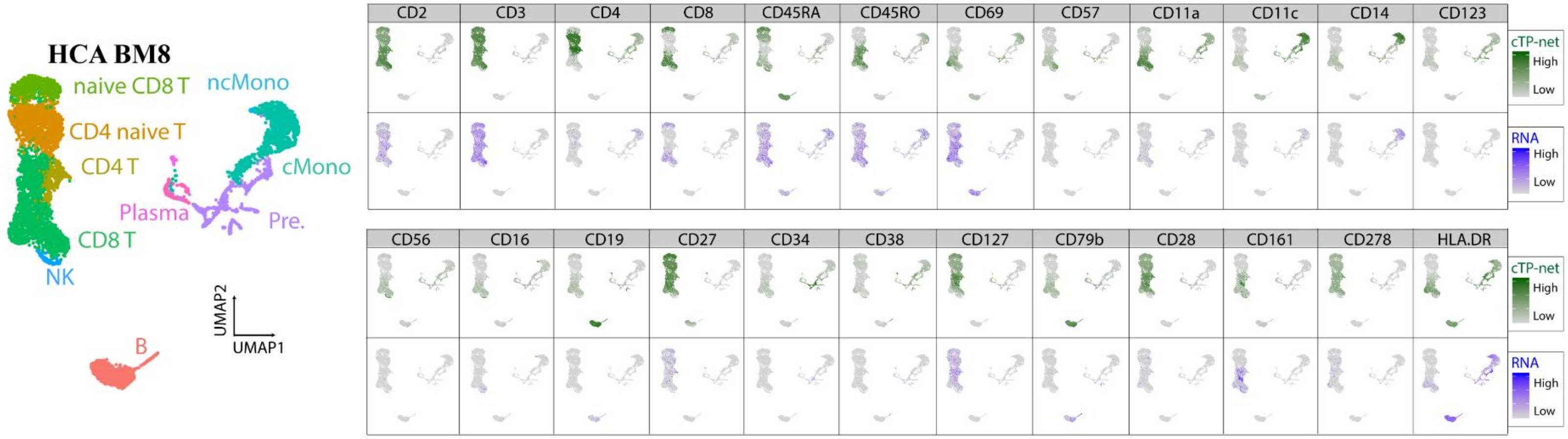
HCA BM8
naive CD8 T
ncMono
CD4 naive T
CD4 T
cMono
Plasma
Pre.
CD8 T
NK
B
UMAP2
UMAP1
CD2
CD3
CD4
CD8
CD45RA
CD45RO
CD69
CD57
CD11a
CD11c
CD14
CD123
cTP-net
High
Low
RNA
High
Low
CD56
CD16
CD19
CD27
CD34
CD38
CD127
CD79b
CD28
CD161
CD278
HLA.DR
cTP-net
High
Low
RNA
High
Low

Supplementary Figure 9
cTP-net prediction on Human Cell Atlas BMMCs by individual.
For each individual, we show (1) t-SNE visualization of HCA BMMCs based on expression. B: B cells; CD4 T: CD4 T cells; CD8 T: CD8 T cells; Mono: Monocyte; NK: Natural killer cells; Pre.: Precursors. (2) cTP-net imputed protein abundance and RNA of its cognate gene across 12 different surface proteins.

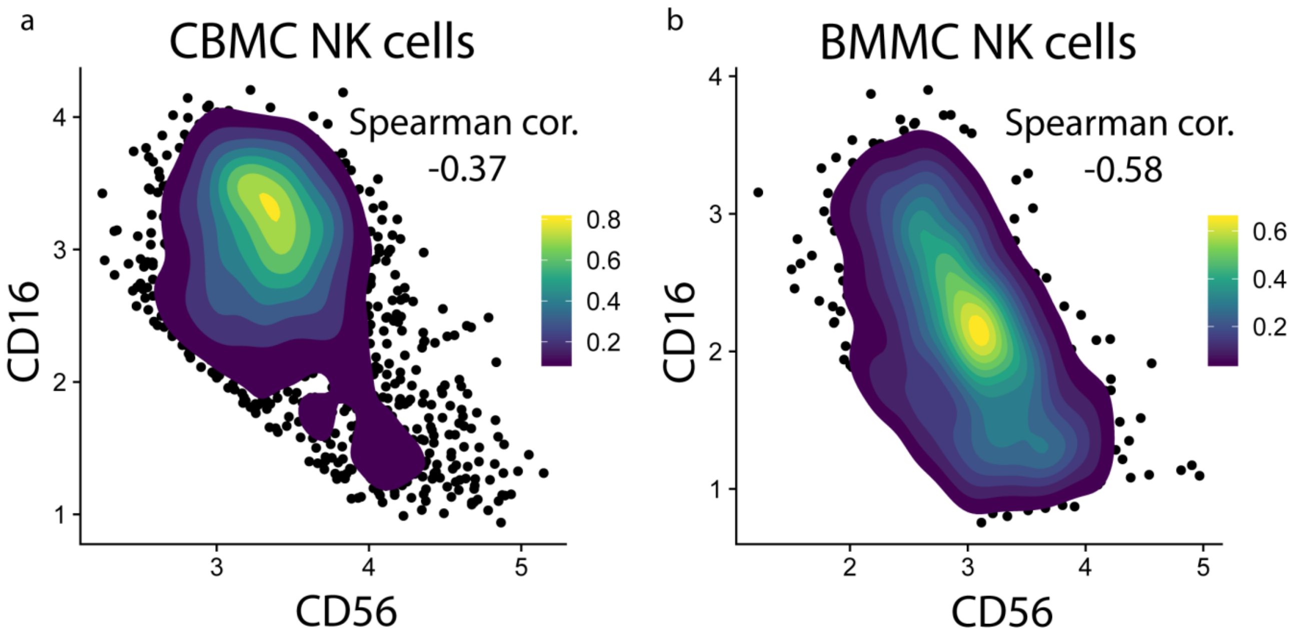

Supplementary Figure 10

Contour plot of cells based on imputed CD56 and CD16 abundance in NK cell populations.

(a) NK cells across all samples from HCA CBMC. (b) NK cells across all samples from HCA BMMC. Strong negative correlation with two subpopulation observed.

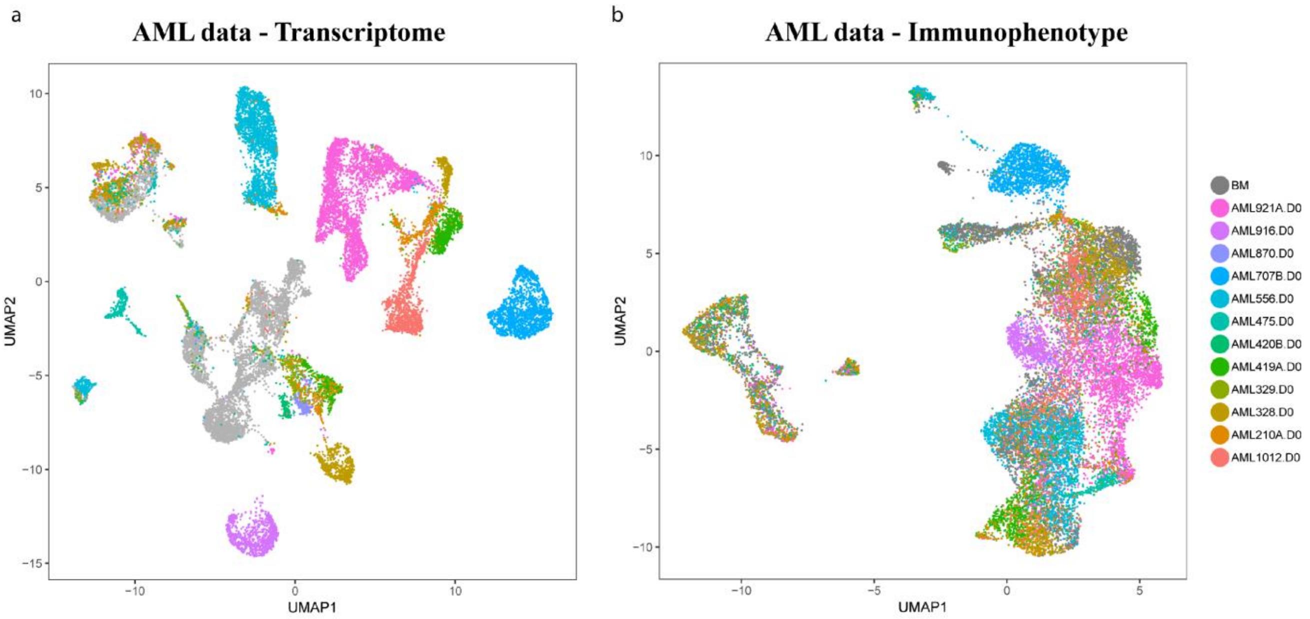

Supplementary Figure 11

UMAP plots of AML data set, colored by samples.

(a) Dimension reduction on transcriptome (RNAs). (b) Dimension reduction on imputed surface proteins.

Supplementary Note. cTP-net models tested on CITE-seq CBMC data sets

Supplementary Table 5 illustrates the different models we have tested. The first column indicates the differences to the finalized models, while the second column shows the correlation of the predicted protein abundance to the true protein abundance in the holdout setting on CITE-seq CBMC data set. As shown by Supplementary Table 5, missing any component of the final model will result in inferior performance.

Supplementary Table 1. Summary table of five data sets analyzed in this study

Data	Technology	Cell population	# of subjects	# of cells	# of genes	# of proteins	# of cell types
**CITE-PBMC**	CITE-seq	PBMC	1	7667	13517	10	8
**CITE-CBMC**	CITE-seq	CBMC	1	8005	14505	10	12
**REAP-PBMC**	REAP-seq	PBMC	1	4326	10811	10	NA
**CITE-BMMC**	CITE-seq	BMMC	1	33455	17009	25	NA
**HCA-CBMC**	10x	CBMC	8	260,000	12611	NA	NA
**HCA-BMMC**	10x	BMMC	8	270,000	12611	NA	NA

Supplementary Table 2. Cell type summary of CITE-seq data sets

Data	Cell types
**CITE-PBMC**	B, CD8 T-1, CD4 T, NK, DC, CD14+CD16+ Mono, CD14-CD16+ Mono, CD8 T 2
**CITE-CBMC**	B, CD8 T, CD4 T, NK, DC, CD14+ Mono, CD16+ Mono, pDC, CD34+, Eryth, Unknown

Supplementary Table 3. Top 20 highest influence score genes for each protein in CITE-PBMC data set

CD3	CD4	CD8	CD2	CD45RA	CD57	CD16	CD14	CD11c	CD19
CD3D	*CD8B*	*CD8B*	*CCL5*	*KLRB1*	*NUDT6*	*CHL1*	*C1orf115*	*CFD*	*CCL5*
IL7R	*CD8A*	*CD8A*	*IL7R*	*CCL5*	*MZT2A*	*RP11-242C19.2*	*PEAK1*	*MAL*	*CD8B*
CD8B	*RP11-291B21.2*	*CCL5*	*RP4-539M6.22*	*EIF1AX*	*ATP2A2*	*GCSH*	*ALDH7A1*	*ANKRD36C*	*RN7SL600P*
FCER1G	*CCL5*	*TRDC*	*LTV1*	*CD7*	*IQCE*	*NRL*	*CYBB*	*BLOC1S3*	*MYO1D*
TRDC	*NCR3*	*RP11-291B21.2*	*RP11-452L6.5*	*TST*	*PKNOX1*	*DBF4*	*ISYNA1*	*IGLL5*	*HSF2*
AKR7A2	*KLRB1*	*CHMP7*	*FBXO10*	*MFSD7*	*FBXW8*	*SPHK2*	*LMAN1*	*RP11-159G9.5*	*AC142528.1*
HELLS	*DDIT3*	*ZAP70*	*LINC00384*	*ZFAS1*	*CTA-217C2.1*	*CDKL1*	*FAM162A*	*ARMCX1*	*DNAJA3*
ALG10	*CTD-2547L16.1*	*BMP8B*	*ACAP2*	*TAPSAR1*	*CNOT11*	*TIMM21*	*SLC4A7*	*SLC6A16*	*GLB1L*
FGD5-AS1	*C18orf25*	*FAH*	*PPCDC*	*PLEKHF1*	*HSD17B4*	*MRPS18C*	*MIER3*	*LRRC16A*	*LIMD2*
COMMD7	*FPGT*	*AC009299.3*	*ANKRD39*	*CTBP1-AS2*	*CLEC4E*	*C7orf43*	*SLC11A2*	*TRAF1*	*DTX3L*
CTA-292E10.8	*NETO2*	*CMKLR1*	*AIM2*	*CYP27A1*	*FAM98C*	*GORASP2*	*ZAP70*	*PABPN1*	*PTCD2*
ZC2HC1A	*GDAP1*	*ENTPD1*	*TTLL12*	*MAN1A2*	*PRMT1*	*CTD-2555C10.3*	*MAP4*	*ADM*	*LPAR1*
INADL	*CSTF1*	*PIK3CA*	*GABBR1*	*FAM115C*	*SLC25A11*	*LEPROT*	*TTTY15*	*KIAA0319L*	*ZNF649*
SHISA4	*RP11-159H10.3*	*WDR7*	*DCUN1D4*	*CST3*	*TCEANC2*	*RUSC1*	*HS1BP3*	*MRPL4*	*HLA-DRB5*
DCAF4	*RP11-451M19.3*	*HEG1*	*CPD*	*NAIF1*	*LCTL*	*POLR2L*	*PRPSAP1*	*NDRG1*	*LIN54*
HPGDS	*ENTPD1-AS1*	*NPAT*	*RAPGEFL1*	*RP11-83N9.5*	*CAPN1*	*RP11-85A1.3*	*ZBTB38*	*FAM63A*	*USP32*
PACSIN1	*SLC4A10*	*7-Sep*	*U91328.20*	*FCGR3A*	*VPS26A*	*FKBP7*	*PIK3R1*	*RPL34*	*AIM2*
ARID4B	*FAAH2*	*CDT1*	*EIF4H*	*CCDC163P*	*ECHS1*	*RNF24*	*PIGG*	*FAM118B*	*SLC12A7*
ATP11A	*AP5B1*	*QRICH1*	*AC073115.7*	*POLR1C*	*FLVCR1-AS1*	*TBXAS1*	*DESI2*	*UBQLN4*	*ZNF671*
RN7SL521P	*DHPS*	*AP2M1*	*RP11-4O1.2*	*PRSS35*	*RP11-421L21.2*	*WDR83*	*SIRT5*	*FKBP15*	*TNNI2*

Supplementary Table 4. Gene set enrichment analysis on cell-immunophenotype pairs that cTP-net predict well in CITE-PBMC data set

Surface protein	Cell type	GO pathways
CD45RA	CD14-CD16+Mono	GO_CATABOLIC_PROCESS
		GO_PROTEIN_LOCALIZATION
		GO_REGULATION_OF_CELLULAR_COMPONENT_BIOGENESIS
		GO_CELLULAR_RESPONSE_TO_STRESS
		GO_CELLULAR_RESPONSE_TO_DNA_DAMAGE_STIMULUS
		GO_RNA_BINDING
		GO_ESTABLISHMENT_OF_LOCALIZATION_IN_CELL
		GO_CELL_CYCLE
		GO_SINGLE_ORGANISM_BIOSYNTHETIC_PROCESS
		GO_CELLULAR_MACROMOLECULE_LOCALIZATION
CD11c	CD14-CD16+Mono	GO_CELLULAR_RESPONSE_TO_STRESS
		GO_NEGATIVE_REGULATION_OF_GENE_EXPRESSION
		GO_POSITIVE_REGULATION_OF_BIOSYNTHETIC_PROCESS
		GO_POSITIVE_REGULATION_OF_GENE_EXPRESSION
		GO_CELL_CYCLE
		GO_POSITIVE_REGULATION_OF_PROTEIN_METABOLIC_PROCESS
		GO_NEGATIVE_REGULATION_OF_NITROGEN_COMPOUND_METABOLIC_PROCESS
		GO_CYTOSKELETON
		GO_CHROMOSOME
		GO_ENZYME_BINDING
CD45RA	CD8 T 2	GO_ENZYME_BINDING
		GO_RNA_BINDING
		GO_RIBONUCLEOPROTEIN_COMPLEX
		GO_REGULATION_OF_TRANSCRIPTION_FROM_RNA_POLYMERASE_II_PROMOTER
		GO_CELL_CYCLE
		GO_RNA_PROCESSING
		GO_POSITIVE_REGULATION_OF_BIOSYNTHETIC_PROCESS
		GO_CYTOSKELETON
		GO_RIBONUCLEOTIDE_BINDING
		GO_POSITIVE_REGULATION_OF_GENE_EXPRESSION
CD45RA	CD4 T	GO_REGULATION_OF_IMMUNE_SYSTEM_PROCESS
		GO_IMMUNE_SYSTEM_PROCESS
		GO_VACUOLE
		GO_SMALL_MOLECULE_METABOLIC_PROCESS
		GO_ORGANONITROGEN_COMPOUND_METABOLIC_PROCESS
		GO_ESTABLISHMENT_OF_LOCALIZATION_IN_CELL
		GO_POSITIVE_REGULATION_OF_MULTICELLULAR_ORGANISMAL_PROCESS
		GO_ENDOPLASMIC_RETICULUM

		GO_REGULATION_OF_TRANSCRIPTION_FROM_RNA_POLYMERASE_II_PROMOTER
		GO_PROTEIN_LOCALIZATION
**CD11c**	**CD14+CD16+ Mono**	GO_POSITIVE_REGULATION_OF_GENE_EXPRESSION
		GO_DNA_REPLICATION
		GO_POSITIVE_REGULATION_OF_MOLECULAR_FUNCTION
		GO_POSITIVE_REGULATION_OF_BIOSYNTHETIC_PROCESS
		GO_SINGLE_ORGANISM_BIOSYNTHETIC_PROCESS
		GO_DNA_DEPENDENT_DNA_REPLICATION
		GO_PHOSPHATE_CONTAINING_COMPOUND_METABOLIC_PROCESS
		GO_CELL_JUNCTION
		GO_CYTOKINE_RECEPTOR_BINDING
		GO_ORGANONITROGEN_COMPOUND_BIOSYNTHETIC_PROCESS
**CD45RA**	**DC**	GO_NEGATIVE_REGULATION_OF_NITROGEN_COMPOUND_METABOLIC_PROCESS
		GO_POLY_A_RNA_BINDING
		GO_CHROMOSOME_ORGANIZATION
		GO_REGULATION_OF_DNA_METABOLIC_PROCESS
		GO_RNA_BINDING
		GO_MACROMOLECULAR_COMPLEX_BINDING
		GO_PHOSPHATE_CONTAINING_COMPOUND_METABOLIC_PROCESS
		GO_NEGATIVE_REGULATION_OF_GENE_EXPRESSION
		GO_ESTABLISHMENT_OF_LOCALIZATION_IN_CELL
		GO_DNA_METABOLIC_PROCESS
**CD11c**	**DC**	GO_ENZYME_BINDING
		GO_RIBONUCLEOTIDE_BINDING
		GO_ESTABLISHMENT_OF_LOCALIZATION_IN_CELL
		GO_NEGATIVE_REGULATION_OF_PROTEIN_METABOLIC_PROCESS
		GO_IMMUNE_SYSTEM_PROCESS
		GO_ORGANONITROGEN_COMPOUND_BIOSYNTHETIC_PROCESS
		GO_PHOSPHATE_CONTAINING_COMPOUND_METABOLIC_PROCESS
		GO_PHOSPHORYLATION
		GO_NEGATIVE_REGULATION_OF_PROTEIN_MODIFICATION_PROCESS
		GO_TRANSFERASE_ACTIVITY_TRANSFERRING_PHOSPHORUS_CONTAINING_GROUPS

Supplementary Table 5. Summary table of different cTP-net models

**Differences to the finalized model**	**Correlation**
Without SAVER-X denoising, without MB structure	0.961±0.0004
Without MB structure	0.968±0.0005
Without SAVER-X denoising	0.959 ±0.0005
L2 loss	0.969±0.0002
Set bottle neck layer to 256 nodes (128 in final model)	0.968±0.0003
Set bottle neck layer to 64 nodes (128 in final model)	0.968±0.0003
With additional shared layers	0.969±0.0004
With SeLU activation function	0.966±0.0002
With Dropout layer between layer1 and layer2	0.966±0.001
Exclude genes corresponding to targeted proteins	0.967±0.0001
**Final model**	**0.970**±0.0003

Supplementary Table 6. List of surface proteins and corresponding genes

Surface protein	Corresponding gene
CD3	*CD3D,CD3E,CD3G,CD247*
CD4	*CD4*
CD8	*CD8A,CD8B*
CD45RA	*PTPRC*
CD56	*NCAM1*
CD2	*CD2*
CD16	*FCGR3A*
CD11c	*ITGAX*
CD14	*CD14*
CD19	*CD19*
CD34	*CD34*
CD57	*B3GAT1*
CD11a	*ITGAL*
CD123	*IL3RA*
CD127	*IL7R*
CD161	*KLRB1*
CD27	*CD27*
CD278	*ICOS*
CD28	*CD28*
CD38	*CD38*
CD45RO	*PTPRC*
CD69	*CD69*
CD79b	*CD79B*
HLR.DR	*HLA-DRA,HLA-DRB1,HLA-DRB5*
